# Factors influencing Chinese cultural identity among new generations of ethnic Chinese in Singapore, Malaysia, and Thailand: a comparative Study

**DOI:** 10.3389/fpsyg.2026.1908594

**Published:** 2026-09-09

**Authors:** Bo Miao, Ye Sun, Haowei Tao

**Affiliations:** 1 School of International Communication and Arts, Hainan University, Haikou, China; 2 School of Culture and Communication, Capital University of Economics and Business, Beijing, China; 3 Department of International Politics, University of International Relations, Beijing, China

**Keywords:** Chinese content exposure, Chinese diaspora, Chinese language proficiency, cultural identity, screen size

## Abstract

**Introduction:**

The cultural identity of overseas Chinese populations, particularly new generations born and raised in their host societies, has become an increasingly important topic in globalization research. Previous studies have largely focused on Western immigrant populations, with limited attention to non-Western, non-migrant communities such as new-generation ethnic Chinese in Southeast Asia. Moreover, prior research has tended to examine language and media factors in isolation. To address these gaps, this study proposes an integrative model examining how Chinese content exposure, large screen preference, visits to China, and Chinese language proficiency shape Chinese cultural identity (CCI) among new generations of ethnic Chinese in Singapore, Malaysia, and Thailand (SMT).

**Methods:**

A self-report questionnaire survey was conducted through an online panel platform, yielding 1,298 valid responses from second-generation or beyond ethnic Chinese across the three countries. One-way ANOVA, hierarchical regression, and mediation analysis using the PROCESS macro with 5,000 bootstrap resamples were employed to test the proposed hypotheses.

**Results:**

Malaysian new-generation ethnic Chinese exhibited the highest CCI levels, followed by Singapore, with Thailand the lowest. Chinese content exposure and visits to China emerged as the two factors most consistently and positively associated with CCI across all three countries. Chinese language proficiency was significantly associated with CCI only in Malaysia, while large screen preference showed inconsistent associations. Mediation analyses revealed that Chinese content exposure mediated the relationships between visits to China and CCI, and between Chinese language proficiency and CCI, across all three countries.

**Discussion:**

These findings underscore the importance of media consumption and direct cultural engagement as key pathways to CCI. The cross-national variations highlight the need for country-specific approaches to understanding diaspora cultural identity. This study contributes to the literature by integrating media and personal factors within a comparative three-country framework, offering a more comprehensive understanding of how CCI is shaped among new-generation overseas Chinese in Southeast Asia.

## 1 Introduction

With the rapid pace of globalization, the development of cultural identity has become a key focus in social science research. Especially in pluralistic societies, individuals must balance the preservation of their traditional cultural values with the need to meet the dominant cultural norms, forming a “hybrid cultural identity” (Belgrade et al., 2022; Ting et al., 2024). This is also true for the new generations of overseas Chinese, those who have Chinese ancestry but no longer hold Chinese citizenship, and who were born and raised overseas (Zheng et al., 2025). For example, new-generation Malaysian Chinese tend to prioritize integration into the national culture (Tan, 1997), yet they still maintain a degree of identification with traditional Chinese culture (Peng and Xie, 2022). In this study, Chinese cultural identity (CCI) is defined as individuals’ cognitive, emotional, and behavioral attachment to the cultural heritage of China (Karjalainen, 2020; Zheng et al., 2025), including festivals, ethical values, and artistic traditions, rather than to the People’s Republic of China as a political entity, or to a generalized pan-ethnic Chineseness. Since the new generations of ethnic Chinese were born and raised as citizens of their respective host societies, their relationship to “Chineseness” may be negotiated through different channels, such as heritage language practices, media consumption, and cultural participation (Chang and Curdt-Christiansen, 2026), rather than through direct migratory experience or ongoing political affiliation with China. This distinct pathway implies that the determinants of their CCI are likely more complex and may differ substantially from those of first-generation immigrants. Hence, it is essential to systematically examine which factors strengthen or weaken CCI among this cohort.

To date, existing studies on this issue have largely converged around three perspectives: Firstly, the heritage language perspective draws primarily on Fishman’s theory of language maintenance (Fishman, 1991). Within this framework, language is understood not merely as a communicative tool, but as an instrument through which cultural knowledge and cultural identity are reproduced across generations. Heritage language retention is thus intimately linked to the maintenance of cultural belonging (He, 2006). Studies have shown that heritage language proficiency is often associated with ethnic cultural attachment among diaspora communities (Zhou and Liu, 2023; Liu et al., 2025; Qian, 2026). However, research on new-generation Chinese Cambodians has found no significant correlation between language inheritance and CCI (Zhang and An, 2025), indicating that findings are not always consistent. This variation may be due to differences in sociopolitical, historical, and educational contexts across countries. To date, most studies have focused on single-country cases, largely in Western settings, and systematic comparative research across Southeast Asian countries remains limited.

Secondly, the media consumption perspective investigates how exposure to China-related content reinforces CCI (Loi, 2023). Studies have found that Malaysian Chinese who consume more Chinese-language media exhibit stronger CCI (Peng and Xie, 2022; Liu et al., 2023). In recent years, digital media consumption has become a key driver of cultural continuity for new-generation ethnic Chinese. Given the unprecedented penetration of Chinese digital platforms in Southeast Asia (Peng, 2025), this generation has greater access to China-related content. Apps like WeChat provide diasporic users with digital tools to sustain imaginaries of Chinese communities and strengthen their sense of cultural belonging (Pyo and Gu, 2023). Nevertheless, existing research has largely focused on media content or platforms, with relatively little attention to the physical materiality of media devices. Moreover, most studies remain single-country case analyses, and cross-national comparative work is still scarce in this strand of literature.

Thirdly, beyond media-related factors, the psychological perspective applies social identity theory to examine how self-esteem, perceived discrimination, and bi-cultural efficacy may shape CCI (Schwartz et al., 2006; Zheng et al., 2025). Social identity theory posits that individuals derive part of their identity from the social groups to which they belong and are motivated to maintain a positive social identity (Tajfel et al., 1986). For diaspora populations, this means that the strength of cultural identification is shaped by the accessibility and salience of cultural group membership, which in turn is influenced by cultural engagement behaviors (Xie et al., 2024; Lyu et al., 2026). Studies in this tradition contribute valuable insights into individual-level psychological mechanisms. However, they rarely incorporate media factors, leaving the interplay between cognitive processes and media engagement unexplored.

Overall, four relatively under-explored areas can be identified in the current body of research: (1) Previous studies have tended to focus predominantly on Western immigrant populations, while non-Western, non-migrant communities such as the new generations of ethnic Chinese in Southeast Asia have received relatively less systematic attention. (2) Although prior research has highlighted the role of China-related media in shaping CCI, the material dimension of media, such as the physical affordances of screen size, has received relatively limited empirical scrutiny (Siles and Boczkowski, 2012). (3) Cross-national comparative studies that could reveal the heterogeneity of Chinese diaspora communities across different Southeast Asian countries remain scarce. Most of the existing studies are based on single-country case studies. (4) There is a relative lack of integrative models that simultaneously account for the multiple pathways through which Chinese language proficiency, inter-group contact with the host society, and media consumption jointly influence CCI among these new generations.

This paper advances the existing literature in four corresponding ways: (1) It specifically targets the new generations of overseas Chinese in Singapore, Malaysia, and Thailand (SMT), which have received less systematic examination compared to first-generation migrants or Western diaspora populations. (2) By integrating media materiality with media content exposure, this study responds to the call for a “texto-material” perspective (Siles and Boczkowski, 2012) and tests the influences of the physical affordances of screens on CCI alongside the effects of content. (3) It introduces a comparative three-country design, which allows for cross-national comparisons and helps capture the diversity of CCI in Southeast Asia. (4) This study develops an integrated analytical framework that simultaneously incorporates media factors (including both content and material dimensions), personal factors (namely visits to China and Chinese language proficiency), thereby offering a more comprehensive understanding of how CCI is constructed among new generations of ethnic Chinese in SMT.

## 2 Literature review and hypotheses

### 2.1 The CCI among new generations of ethnic Chinese in SMT

New generations of ethnic Chinese in SMT exhibit distinctive patterns of CCI. Comparative research between Malaysia and Thailand reveals that Malaysian Chinese new generations exhibit stronger “ancestral memory.” 60.7% of Malaysian Chinese respondents selected “I am first and foremost a citizen of my country of residence, but I am fully aware of my Chinese cultural identity,” demonstrating the “dual strength” in both national identity and CCI among Malaysian Chinese new generations (Yu and Yang, 2006; Yang and Yu, 2007). By contrast, 41.7% of Thai respondents selected “I am a citizen of my country of residence, no different from indigenous residents, having Chinese cultural identity does not matter” (Yang and Yu, 2007). This difference may be attributed to the divergent historical, political, and economic trajectories of Malaysia and Thailand. Malaysia was once a British colony, and during the colonial period, the British administration implemented a “divide and rule” policy. This policy, which segregated the Chinese community and encouraged a degree of self-governance, had the unintended effect of reinforcing Chinese cultural identity (Yi and Sun, 2022). After Malaysia gained independence, the country has continued to implement policies that give preference to the Malay community. The “Malay-first” policies have also contributed to situations in which new-generation Malaysian Chinese may face unequal treatment in areas such as education and employment. Such disparities in political and economic spheres might make them more inclined to seek identification with Chinese culture (Yi, 2020). In contrast, Thailand is the only country in Southeast Asia that was never colonized by a Western power. Its relative policy autonomy allowed it to introduce and enforce a series of assimilation policies targeting the Chinese community (Yi and Sun, 2022). For instance, the Thai government mandated Thai-language education, and also implemented a surname-change policy, requiring ethnic Chinese to adopt Thai surnames. The cumulative effect of these policies contributed to a rupture in Chinese-language education in Thailand, ultimately resulting in a new generation of ethnic Chinese who, to a large extent, cannot speak Chinese and tend not to identify with Chinese culture (Yi and Sun, 2022). For Singapore, it was separated from the Federation of Malaysia in 1965 and became an independent sovereign state. However, research on CCI among the new generations of ethnic Chinese in Singapore is relatively scarce. Singapore presents a unique case where ethnic Chinese constitute approximately 74% of the resident population, making them the demographic majority rather than a minority (Ministry of Health Singapore, 2024). The state’s bilingual policy promotes Chinese as a designated “mother tongue” while privileging English as the working language, creating a highly institutionalized yet somewhat instrumental environment for Chinese cultural transmission (Chua, 1995). What is the current state of CCI among the new generations of ethnic Chinese in Singapore? How do the patterns of CCI compare across the new generations in SMT? And what factors may influence CCI among these populations? These are key questions that deserve systematic examination.

To answer these questions, we concentrate on the CCI of new generations of ethnic Chinese in SMT. Our rationale for this focus rests on three considerations. First, together, these three nations host one of the largest Chinese diaspora populations in the world. In Singapore, ethnic Chinese constitute the overwhelming majority of the population (Ministry of Health Singapore, 2024). In Malaysia, while the Chinese constitute a minority, their absolute numbers remain substantial, amounting to approximately 6.9 million, representing roughly 22.2% of the national population (Department of Statistics Malaysia, 2026). Thailand presents a more complex picture due to centuries of intermarriage and assimilation, but approximately 7 million individuals claim either full or partial Chinese ancestry (Minority Rights Group International, 2018). These three countries are major settlements of overseas Chinese. Second, unlike earlier generations who maintained direct familial ties to ancestral villages in China, contemporary Chinese youth in SMT were born and raised as full citizens of their respective nations. Many are now third, fourth, or even fifth generation descendants, to whom China is more of an imagined homeland, rather than a lived memory. Their prolonged lack of direct ties to China raises an important question about how CCI can be sustained without personal migratory experience. Third, the transformation of CCI among Chinese youth in SMT may not be fully explained by existing frameworks such as family socialization (Zhang et al., 2025), clan association participation (Chen, 2022; Li, 2024), or heritage language education (Gao, 2025). While these remain relevant, digital media consumption has emerged as an increasingly important driver of cultural continuity for this generation. Thus, understanding how media and personal factors may shape their CCI is of particular importance.

### 2.2 Media factors and CCI

For media factors, this study adopts the “texto-material” perspective (Siles and Boczkowski, 2012) by integrating media content exposure with media materiality. This perspective resonates with the broader “materiality turn” in media studies, which has called for approaches that recognize the entanglement of symbolic meaning and physical infrastructure in media use. Within the texto-material framework adopted in this study, media factors encompass both the symbolic dimension (namely Chinese content exposure) and the material dimension (namely large screen preference). Chinese content exposure refers to the frequency and extent to which individuals consume China-related media content across various formats, including television programs, films, online videos, and games. Prior research has established that media content serves as an important vehicle for cultural transmission and identity reinforcement among diaspora populations (Peng and Xie, 2022). Studies on Chinese Malaysians have demonstrated that exposure to Cantonese media content is significantly associated with enhanced Chinese cultural identification (Liu et al., 2023). Based on these findings, the present study proposes:

*H1a*: Chinese content exposure is positively associates with CCI among new generations of ethnic Chinese in SMT.

Large screen preference pertains to individuals’ subjective inclination toward using larger-screen devices, such as televisions, cinema screens, over smaller screens like smartphones for media consumption. This preference reflects not merely a technological choice but a material condition that may shape the quality of cultural engagement. Research on screen size and viewer experience has demonstrated that larger screens tend to elicit higher levels of immersion compared to smaller screens, suggesting that screen size may affect how viewers engage with content (Kim and Sundar, 2016; Cho, 2023). In the context of diaspora media consumption, the size of screens may influence the depth of cultural engagement and, consequently, the strength of cultural identification. However, the direct association between screen preference and cultural identity among diaspora populations has received relatively limited empirical attention. The present study therefore poses the following research question:

*RQ1*: Does large screen preference directly associates with CCI?

### 2.3 Personal factors and CCI

Beyond media-related factors, individuals’ personal engagement with their heritage culture, through behavioral participation or linguistic competence, may constitute another important set of predictors of CCI. A growing body of research has examined the relationship between visits to China and cultural identity among overseas Chinese populations (Wang et al., 2024; Li et al., 2025). These studies generally suggest that physical travel to the ancestral homeland serves as an important mechanism through which cultural identity can be reinforced. However, it has also been observed that visits to the ancestral homeland do not uniformly strengthen emotional and cultural ties to China (Tie et al., 2015). Taken together, the existing literature tends to suggest a positive association between visits to China and cultural identity among overseas Chinese populations, while also acknowledging the complexity and variability of this relationship. For the new generations of ethnic Chinese in SMT, visits to China may contribute to developing or strengthening their CCI in ways that are not possible through media consumption or heritage language learning alone. Based on this body of evidence, the present study proposes:

*H2a*: Visits to China is positively associates with CCI among new generations of ethnic Chinese in SMT.

In addition, the relationship between heritage language proficiency and cultural identity has been extensively documented in diaspora research. Studies have shown that heritage language proficiency serves as a key marker of cultural identity and a conduit for cultural transmission (Wang, 2023; Zhou and Liu, 2023). Among overseas Chinese communities, language is not merely a communicative tool but also a carrier of cultural values and affective ties. Research on Thailand and Malaysian Chinese youth has also shown that exposure to the Chinese language is associated with a sense of closeness to ancestral heritage and identification with Chinese culture (Yang and Yu, 2007). These findings suggest that linguistic competence in the heritage language is positively associated with cultural identification. Accordingly, we propose the following hypothesis:

*H2b*: Chinese language proficiency is positively associates with CCI among new generations of ethnic Chinese in SMT.

### 2.4 The mediating roles of Chinese content exposure

Beyond identifying the direct effects of media and personal factors on CCI, it is also important to consider the potential mediating pathways through which these factors may exert their influence. Specifically, media consumption, particularly exposure to Chinese content, may serve as a mediating mechanism that translates media material conditions, behavioral engagements, and linguistic competencies into cultural identification. First, large screen preference reflects not merely a technological choice but a material condition that may shape the quality and quantity of cultural content consumption. Existing findings suggest that larger screens tend to be associated with greater immersion, stronger presence, and more favorable emotional responses to media content (Kim and Sundar, 2016; Cho, 2023). The enhanced visual richness, spatial presence, and emotional immersion afforded by larger screens may make Chinese films, television dramas, variety shows, and other cultural content more appealing and accessible, which in turn may be positively associated with CCI. Based on this reasoning, we propose H3a:

*H3a*: Chinese content exposure mediates the relationship between large screen preference and CCI.

Furthermore, visits to China may also be positively associated with Chinese content exposure. Individuals who actively maintain connections with their heritage culture may be more likely to seek out and engage with Chinese media content as a natural extension of their cultural engagement. Visits to China may expose individuals to Chinese media recommendations, cultural events, and shared content, thereby increasing their consumption of Chinese cultural materials and in turn enhancing their CCI. Thus, H3b is suggested:

*H3b*: Chinese content exposure mediates the relationship between visits to China and CCI.

Finally, Chinese language proficiency may be positively associated with Chinese content exposure. Language proficiency lowers the barriers to media consumption, enabling deeper and more frequent engagement with Chinese cultural content. Conversely, limited Chinese proficiency may restrict individuals to content available in their dominant language, thereby reducing their exposure to Chinese cultural materials. That is, Chinese content exposure may play mediating roles in the relationship between language proficiency and CCI:

*H3c*: Chinese content exposure mediates the relationship between Chinese language proficiency and CCI.

**FIGURE 1:**
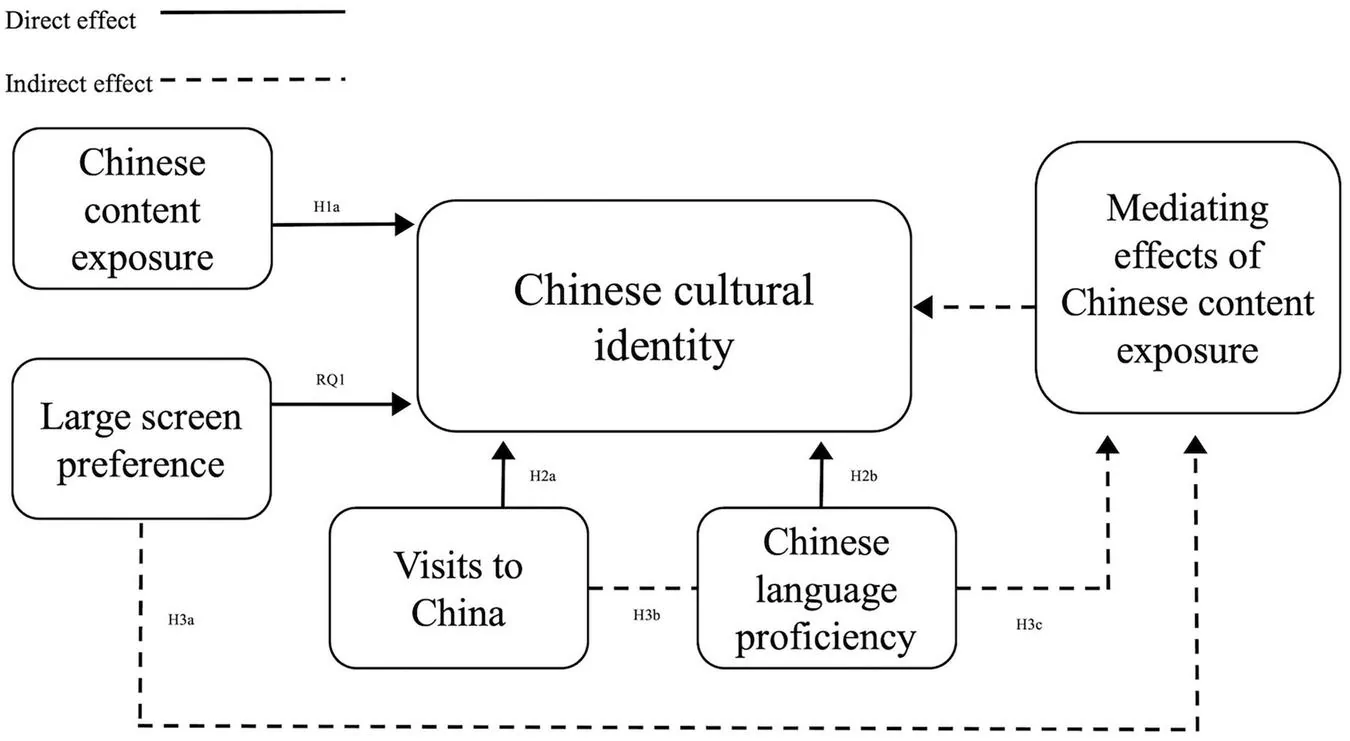
Research model.

Based on the discussions above, we propose a theoretical model as shown in Figure 1.

## 3 Research methods

To explore the proposed model, this study used a self-report questionnaire survey to measure the Chinese content exposure, large screen preference, visits to China, Chinese language proficiency, and CCI. The study was reviewed and approved by the Institutional Review Board, School of Culture and Communication, Capital University of Economics and Business (Approval No. 20260429001). All participants provided signed consent for participation.

### 3.1 Sample

The questionnaire survey was conducted in two stages. First, a pretest was carried out in February 2026. It involved 36 young ethnic Chinese from SMT recruited via snowball sampling, and its purpose was to assess the questionnaire’s readability and content validity. Based on feedback, the final questionnaire was developed. Second, the main survey was outsourced to Questionnaire Star in March 2026.

The target sample of this study was drawn from the proprietary sample panel of the “Surveymars” platform, a global sample collection channel operated by Questionnaire Star. The platform is headquartered in Hong Kong, with servers distributed across Germany, the United States, and Singapore. Its sample panel covers 148 countries and regions worldwide, with registered users widely distributed across different geographic regions, age groups, occupations, and income levels, providing a diverse sample base for the research. Due to commercial confidentiality, the platform only discloses the age distribution of overseas Chinese in its sample panel from SMT, while other information is not disclosed. To date, no government agency or research institution has published demographic statistics specifically for the new generations of ethnic Chinese in Singapore, Malaysia, and Thailand. Moreover, demographic statistics for Chinese-Thais have not been disclosed by the Thai government or other research institutions. Consequently, we can only assess the representativeness of our sample by comparing the age distribution of the Surveymars sample panel with the available official age distributions for the ethnic Chinese populations of Singapore and Malaysia (Department of Statistics Malaysia, 2026; National Population and Talent Division, 2024). Table 1 presents the relevant data. The comparison between the sample panel and the official demographic benchmarks reveals a noticeable skew: the panel over-represents respondents aged 25 to 44 while substantially under-representing those aged 45 and above. This discrepancy, however, is not an idiosyncratic flaw but rather a well-documented characteristic of non-probability online access panels, which can be attributed to the coverage bias inherent in such panels. Any survey relying on an online panel tends to under-sample older age groups, as their presence in the sampling frame is structurally constrained (Couper, 2008). Given that our research explicitly targets new generations of ethnic Chinese, the relative over-representation of the 25–44 age bracket aligns with the study’s primary focus. To mitigate this bias, demographic variables were included as statistical controls in the model, and this limitation is acknowledged in the final section.

**TABLE 1:** Comparison of age distribution between Surveymar’s overall sample panel and official population benchmarks for Singaporean and Malaysian Chinese.

Countries	18–24	25–29	30–34	35–39	40–44	45 or above
Age distribution of the sample panel						
Singapore	11.8%	24.5%	24.0%	16.4%	12.7%	11.6%
Malaysia	12.3%	27.3%	23.7%	15.5%	12.0%	9.2%
Thailand	9.2%	21.6%	22.3%	16.2%	14.2%	16.5%
**Countries**	**15–24**	**25–29**	**30–34**	**35–39**	**40–44**	**45 or above**
Age distribution of Singaporean Chinese that above 15	11.6%	7.0%	7.7%	7.5%	7.5%	58.7%
Age distribution of Malaysian Chinese that above 15	18.3%	8.3%	9.0%	9.7%	10.2%	44.5%

Before conducting this study, we established clear sample screening criteria based on the research objectives, including respondents of second-generation or above ethnic Chinese from Singapore, Malaysia, and Thailand, with additional provisions regarding geographic distribution. The platform conducted preliminary screening and matching within the sample pool based on these criteria, thereby constructing a dedicated sampling frame aligned with the study’s target population. After defining the target population, the platform sent questionnaire invitations to eligible potential respondents in the sample pool. To avoid duplicate submissions, the platform enforced restrictions allowing only one questionnaire completion per account and per IP address. To ensure final data quality, the system also excluded invalid questionnaires during the data cleaning phase, those that failed screening questions, had IP addresses not matching the designated countries, or displayed seemingly contradictory responses across consecutive matrix scale questions. A total of 1,884 questionnaires were collected, and after excluding 586 invalid responses, 1,298 valid questionnaires were retained.

The language used in the questionnaire survey was English. English is an official language of Singapore, making it appropriate for the survey in that country. In addition, as Malaysia was once a British colony, Malaysian Chinese tend to have a relatively high level of English proficiency (EF English Proficiency Index, 2025). Therefore, the survey for the new generations of ethnic Chinese in Malaysia was also conducted in English. For Thai Chinese, their English proficiency is comparatively lower (EF English Proficiency Index, 2025), but in order to maintain consistency in the questionnaire and ensure comparability of the data, we also used English for the survey of Thai Chinese new generations. We conducted a pretest to ensure that the English used was relatively simple and easy for Thai Chinese respondents to understand. Nevertheless, we recognize that this may introduce a bias toward Thai Chinese who are more proficient in English, and we acknowledge this limitation in the final section. Moreover, because English proficiency is generally unrelated to Chinese cultural identity, the impact of such sample bias on the findings of this study is probably limited.

All data are stored on the server of the School of Culture and Communication, Capital University of Economics and Business. Table 2 presents the demographic composition of the sample.

**TABLE 2:** Demographic information of our sample (*N* = 1,298).

Variable	Category	Count	Percentage
Nationality	Singapore	424	32.6%
	Malaysia	432	33.3%
	Thailand	442	34.1%
Gender	Male	671	51.7%
	Female	627	48.3%
Age	18–25	203	15.6%
	26–35	595	45.8%
	36–45	381	29.4%
	46–55	105	8.1%
	56 and above	14	1.1%
Education level	Primary school or below	8	0.6%
	Secondary school	76	5.9%
	Vocational education or associate degree	282	21.7%
	Bachelor’s degree	798	61.5%
	Master’s degree	124	9.5%
	Doctorate degree or above	10	0.8%
Ancestral hometown in China	Hainan	140	10.8%
	Guangdong	656	50.5%
	Guangxi	33	2.5%
	Fujian	337	26%
	Others	132	10.2%
Immigrant generation	Second generation	330	25.4%
	Third generation	651	50.1%
	Fourth generation	272	21%
	Fifth generation and above	45	3.5%

### 3.2 Measures

#### 3.2.1 Chinese content exposure

The Chinese content exposure items (assessments ranged from 1 = “Never” to 5 = “Very often”; *min* = 1, *max* = 4.83, *M* = 2.18, *SD* = 0.65, Cronbach’s α = 0.804) were adapted from prior research on media exposure in a changing communication environment (Slater, 2004; de Vreese and Peter, 2016), including exposure to Chinese movies, television dramas, video games, novels, and other China-related media content (Fang and Duff, 2018). The average score of all items represents Chinese content exposure.

#### 3.2.2 Large screen preference

The large screen preference items (assessments ranged from 1 = “Strongly disagree” to 5 = “Strongly agree”; *min* = 1, *max* = 5, *M* = 3.86, *SD* = 0.79, Cronbach’s α = 0.638) were developed based on prior research on device choice, screen size, and mobile information processing (Kim and Sundar, 2014; Al Ghamdi et al., 2015). The measurement items included “I prefer using large screen devices for important information” etc. Also, the average of the measurement items represents large screen preference.

#### 3.2.3 Visits to China

The visits to China items were adapted for the overseas Chinese context. The item design drew on the ethnic behavior and cultural-practice components of the Multi-group Ethnic Identity Measure (Phinney, 1992), as well as research on Chinese American roots tourism and ancestral homeland visits (the “Frequency of coming to China” item was assessed from 1 = “Never” to 5 = “Very often,” the “How many times have you visited China?” item was measured from 1 = “Never” to 4 = “6 times or more”; *min* = 1, *max* = 4.5, *M* = 1.98, *SD* = 0.68, Cronbach’s α = 0.854). Also, the average of the measurement items represents visits to China.

#### 3.2.4 Chinese language proficiency

Chinese language proficiency was measured by two items, including “how long did you attend a Chinese language school” and “how fluent are you in Chinese” (for the “attending a Chinese language school” item, assessment ranged from 1 = “Never” to 6 = “10 years or more”; for the “fluency in Chinese” item, assessment ranged from 1 = “Don’ t know Chinese at all” to 5 = “Very fluent”; *min* = 1, *max* = 5.5, *M* = 2.67, *SD* = 1.25, Cronbach’s α = 0.877). The average of the two items represents the Chinese language proficiency.

#### 3.2.5 Chinese cultural identity

The CCI items (assessments ranged from 1 = “Strongly disagree” to 5 = “Strongly agree”; *min* = 1.27, *max* = 4.64, *M* = 2.56, *SD* = 0.99, Cronbach’s α = 0.944) were developed based on the measurements of identification with Asian culture (Bhugra et al., 1999) and identification with Chinese culture (Liu et al., 2023). The items evaluated participants’ cognition, affection and behavior related to Chinese culture, and the average of the items represents CCI.

## 4 Results

### 4.1 Reliability and validity analysis

We used confirmatory factor analysis (CFA) in AMOS to assess the reliability and validity of the questionnaire. For reliability, as shown in Table 3, the composite reliability (CR) values for the majority of variables exceeded 0.70, suggesting good reliability. Only the CR for “large screen preference” was 0.638, which is above 0.60 and falls within an acceptable range (Hair et al., 2019).

**TABLE 3:** Composite reliability and convergent validity.

Constructs and items	Factor loading	*R* ^2^	Error	CR	AVE
Chinese content exposure				0.832	0.457
Read paper-based novels related to China	0.564	0.318	0.682		
Being fans of Chinese entertainment stars	0.596	0.355	0.645		
Play games related to China	0.667	0.445	0.555		
Watch Chinese movies, TV series, or Variety shows	0.851	0.724	0.276		
Browse news and commentary related to China	0.615	0.378	0.622		
Other content about China	0.721	0.520	0.480		
**Large screen preference**				0.638	0.469
I prefer using large screen devices for important information	0.688	0.473	0.527		
On smartphones, I tend to skim rather than read deeply	0.681	0.464	0.536		
**Visits to China**				0.873	0.776
Frequency of coming to China to visit relatives and friends, conduct business, study, or travel	0.975	0.951	0.049		
Times visited China	0.776	0.602	0.398		
**Chinese language proficiency**				0.889	0.799
Years of attending a Chinese language school	0.908	0.824	0.176		
How fluent are you in Chinese	0.880	0.774	0.226		
**Chinese cultural identity**				0.946	0.612
I am familiar with famous Chinese historical sites such as the Forbidden City.	0.791	0.626	0.374		
I am familiar with Chinese historical figures such as Confucius and Li Bai.	0.803	0.645	0.355		
I understand Chinese traditional festivals such as the Lantern Festival and the Mid-Autumn Festival.	0.784	0.615	0.385		
I believe that inheriting and developing Chinese culture is very important.	0.787	0.619	0.381		
I like Chinese culture.	0.791	0.626	0.374		
I appreciate Chinese culture.	0.784	0.615	0.385		
I am proud of Chinese culture.	0.710	0.504	0.496		
I intend to share Chinese culture with non-Chinese friends.	0.809	0.654	0.346		
I am interested in purchasing Chinese products.	0.803	0.645	0.355		
I am interested in learning more Chinese language or traditional Chinese arts	0.778	0.605	0.395		
I am interested in traveling, studying, or working in China.	0.764	0.584	0.416		

We also examined two types of validity: convergent validity and discriminant validity. Convergent validity was assessed by examining standardized factor loadings and the average variance extracted (AVE). As shown in Table 3, the standardized factor loadings of all items exceeded the recommended threshold of 0.5. Most AVE values were above or close to 0.5, indicating acceptable convergent validity (Chin, 2010).Notably, the AVE values for Chinese content exposure and large screen preference fall slightly below the recommended threshold of 0.5. This may be because the measurement items for Chinese content exposure were selected based on popular Chinese cultural content overseas. Although each item is relevant to Chinese culture, the nature of the various content types differs, which may have reduced the AVE value. And for large screen preference, the relatively low AVE may be attributable to the lack of a well-established and mature scale in the existing literature. Nevertheless, since both AVE values are only marginally below 0.5, the potential influence on the overall findings is likely to be limited. Discriminant validity refers to the degree to which each latent variable is distinct from all other variables in the model (Chin, 2010). Table 4 shows that the square roots of the AVE values (presented on the diagonal of the correlation matrix) are larger than the inter-construct correlations (shown in the off-diagonal entries) for all constructs, indicating adequate discriminant validity.

**TABLE 4:** Discriminant validity.

Variables	CCE	LSP	VTC	CLP	CCI
CCE	0.676	0.685	0.881	0.894	0.782
LSP	−0.153**				
VTC	0.576**	−0.122**			
CLP	0.404**	0.029	0.626**		
CCI	0.552**	−0.011	0.529**	0.469**	

CCE, Chinese content exposure; LSP, Large screen preference; VTC, visits to China; CLP, Chinese language proficiency; CCI, Chinese cultural identity. **indicates significant correlation at the 0.01 level.

### 4.2 Comparative analysis of CCI among new generations of ethnic Chinese in SMT

We used one-way ANOVA in SPSS to compare the differences among the new generations of ethnic Chinese in SMT on various variables. As shown in Table 5, the new generations of ethnic Chinese in SMT demonstrated a moderate-to-high level of CCI (*M* = 2.56, SD = 0.99, Min = 1.27, Max = 4.64). Among the three countries, the new generations of ethnic Chinese in Malaysia reported the highest level of CCI (*M* = 2.99, SD = 1.07, Min = 1.27, Max = 4.64), followed by Singapore (*M* = 2.68, SD = 1.00, Min = 1.27, Max = 4.55), while the group in Thailand exhibited the lowest level (*M* = 2.02, SD = 0.58, Min = 1.27, Max = 4.45).

**TABLE 5:** One-way ANOVA.

Variables	SMT (*N* = 1298)	Singapore (*N* = 424)	Malaysia (*N* = 432)	Thailand (*N* = 442)	ANOVA	
					*f*-value	*p*-value
Chinese content exposure	*M* = 2.18	*M* = 2.27	*M* = 2.38	*M* = 1.90	71.15	0.000
Large screen preference	*M* = 3.86	*M* = 3.92	*M* = 3.90	*M* = 3.77	4.89	0.008
Visits to China	*M* = 1.98	*M* = 2.08	*M* = 2.23	*M* = 1.63	105.43	0.000
Chinese language proficiency	*M* = 2.67	*M* = 2.95	*M* = 3.46	*M* = 1.64	396.33	0.000
Chinese cultural identity	*M* = 2.56	*M* = 2.68	*M* = 2.99	*M* = 2.02	131.49	0.000

Meanwhile, as shown in Table 6, on variables including Visits to China, Chinese language proficiency, and CCI, the mean scores of new generations of ethnic Chinese in Malaysia were significantly higher than those of their counterparts in Singapore and Thailand, and the mean scores of this group in Singapore were also significantly higher than those in Thailand. Regarding Chinese content exposure, no significant difference was found between the Malaysian and Singaporean groups. However, both the Malaysian and Singaporean new generations of ethnic Chinese reported significantly higher levels of Chinese content exposure than their Thai counterparts.

**TABLE 6:** *Post-hoc* comparisons.

Variables	Test for difference of means (Tamhane’s T2)			
	Comparison groups	Mean difference	*Post-hoc p*-value	95% CI
Chinese content exposure	Malaysia-Singapore	0.10	0.073	−0.01, 0.21
	Malaysia-Thailand	0.47	0.000	0.38, 0.57
	Singapore-Thailand	0.37	0.000	0.27, 0.47
Visits to China	Malaysia-Singapore	0.15	0.005	0.04, 0.26
	Malaysia-Thailand	0.60	0.000	0.50, 0.70
	Singapore-Thailand	0.45	0.000	0.35, 0.55
Chinese language proficiency	Malaysia-Singapore	0.51	0.000	0.34, 0.69
	Malaysia-Thailand	1.81	0.000	1.66, 1.97
	Singapore-Thailand	1.30	0.000	1.15, 1.46
Chinese cultural identity	Malaysia-Singapore	0.31	0.000	0.14, 0.48
	Malaysia-Thailand	0.97	0.000	0.83, 1.11
	Singapore-Thailand	0.66	0.000	0.53, 0.80

### 4.3 Media factors associated with CCI

The research hypotheses were examined via hierarchical regression analysis conducted in SPSS and mediation analysis using the PROCESS macro, with 5,000 bootstrap resamples.

As shown in Table 7, for media factors, Chinese content exposure was significantly and positively associated with CCI in the overall SMT sample (*B* = 0.551, 95% CI [0.469, 0.632], SE = 0.041, *p* < 0.01). This positive association was consistently observed across all three countries for Singapore (*B* = 0.681, 95% CI [0.535, 0.826], SE = 0.074, *p* < 0.01), Malaysia (*B* = 0.611, 95% CI [0.457, 0.766], SE = 0.079, *p* < 0.01), and Thailand (*B* = 0.208, 95% CI [0.098, 0.318], SE = 0.056, *p* < 0.01). These findings indicate that among the new generations of ethnic Chinese in SMT, those who engage more frequently with China-related media content tend to exhibit stronger identification with Chinese culture, supporting H1a.

**TABLE 7:** Hierarchical regression analysis predicting CCI.

Variables	SMT (*N* = 1,298)		Singapore (*N* = 424)		Malaysia (*N* = 432)		Thailand (*N* = 442)	
	B and 95% CI	Std. Error	B and 95% CI	Std. Error	B and 95% CI	Std. Error	B and 95% CI	Std. Error
Demographic control variables								
Gender (1 = Male)	0.037 [−0.048, 0.121]	0.043	−0.049 [−0.207, 0.109]	0.080	0.061 [−0.109, 0.230]	0.086	0.070 [−0.028, 0.169]	0.050
Age	−0.060* [−0.110, −0.010]	0.026	0.040 [−0.051, 0.131]	0.046	−0.158** [−0.260, −0.055]	0.052	−0.031 [−0.090, 0.028]	0.030
Education level	−0.006 [−0.071, 0.059]	0.033	−0.031 [−0.162, 0.100]	0.067	−0.019 [−0.147, 0.108]	0.065	0.069 [−0.008, 0.146]	0.039
Immigrant generation	0.002 [−0.053, 0.057]	0.028	0.066 [−0.041, 0.173]	0.055	0.062 [−0.063, 0.187]	0.064	−0.030 [−0.091, 0.031]	0.031
Subjective social status	−0.015 [−0.049, 0.019]	0.017	0.017 [−0.046, 0.08]	0.032	−0.013 [−0.077, 0.051]	0.033	−0.060** [−0.103, −0.017]	0.022
Adjusted *R*^2^	0.019		0.019		0.002		0.024	
Media factors								
Chinese content exposure	0.551** [0.469, 0.632]	0.041	0.681** [0.535, 0.826]	0.074	0.611** [0.457, 0.766]	0.079	0.208** [0.098, 0.318]	0.056
Large screen preference	0.077** [0.022, 0.132]	0.028	0.094 [−0.021, 0.210]	0.059	0.065 [−0.046, 0.177]	0.057	0.039 [−0.018, 0.097]	0.029
**Δ** Adjusted *R*^2^	0.294		0.286		0.269		0.091	
Personal factors								
Visits to China	0.305** [0.216, 0.394]	0.046	0.202* [0.041, 0.363]	0.082	0.360** [0.190, 0.531]	0.087	0.291** [0.175, 0.407]	0.059
Chinese language proficiency	0.158** [0.112, 0.203]	0.023	0.059 [−0.031, 0.149]	0.046	0.135** [0.036, 0.234]	0.050	0.049 [−0.023, 0.121]	0.036
**Δ** Adjusted *R*^2^	0.086		0.019		0.077		0.063	
Total adjusted *R*^2^	0.399		0.324		0.348		0.178	
*F*-value	96.496		23.548		26.531		11.580	
*P*-value	0.000		0.000		0.000		0.000	

**Indicates significant relation at the 0.01 level; *indicates significant relation at the 0.05 level; the values in brackets are 95% confidence intervals.

Regarding large screen preference, the analysis revealed a significant positive association with CCI in the overall SMT sample (*B* = 0.077, 95% CI [0.022, 0.132], SE = 0.028, *p* < 0.01). However, this relationship was not consistently significant across the three country-specific models. In Singapore, the association was not significant (*B* = 0.094, 95% CI [−0.021, 0.210], SE = 0.059, *p* > 0.05). In Malaysia, it was not significant (*B* = 0.065, 95% CI [−0.046, 0.177], SE = 0.057, *p* > 0.05). And in Thailand, it was also not significant (*B* = 0.039, 95% CI [−0.018, 0.097], SE = 0.029, *p* > 0.05). Since the relationship did not achieve statistical significance across all three countries, the direct association between large screen preference and CCI is not consistently supported, providing no conclusive answer to RQ1.

The inclusion of media factors produced substantial increments in explained variance. The Δ Adjusted R^2^ was 0.294 for the overall SMT sample, 0.286 for Singapore, 0.269 for Malaysia, and 0.091 for Thailand, indicating that media-related variables collectively contributed considerably to the explanation of CCI.

### 4.4 Personal factors associated with CCI

Visits to China was significantly and positively associated with CCI in the overall SMT sample (*B* = 0.305, 95% CI [0.216, 0.394], SE = 0.046, *p* < 0.01). This positive association held consistently across all three country-specific models for Singapore (*B* = 0.202, 95% CI [0.041, 0.363], SE = 0.082, *p* < 0.05), Malaysia (*B* = 0.360, 95% CI [0.190, 0.531], SE = 0.087, *p* < 0.01), and Thailand (*B* = 0.291, 95% CI [0.175, 0.407], SE = 0.059, *p* < 0.01). These results suggest that new-generation ethnic Chinese who have made more frequent visits to China tend to report higher levels of CCI, supporting H2a.

Chinese language proficiency showed a significant positive association with CCI in the overall SMT sample (*B* = 0.158, 95% CI [0.112, 0.203], SE = 0.023, *p* < 0.01). However, this relationship was not consistently supported across all three countries. In Singapore, the association was not significant (*B* = 0.059, 95% CI [−0.031, 0.149], SE = 0.046, *p* > 0.05). In Malaysia, it was significant (*B* = 0.135, 95% CI [0.036, 0.234], SE = 0.050, *p* < 0.01). And in Thailand, it was not significant (*B* = 0.049, 95% CI [−0.023, 0.121], SE = 0.036, *p* > 0.05). Given the lack of consistent significance across all three countries, the association between Chinese language proficiency and CCI is not considered consistently established, and H2b is therefore not supported.

The personal factors contributed additional variance to the model, with Δ Adjusted R^2^ values of 0.086 for the overall SMT sample, 0.019 for Singapore, 0.077 for Malaysia, and 0.063 for Thailand. The total adjusted R^2^ for the full model was 0.399 for the overall SMT sample, 0.324 for Singapore, 0.348 for Malaysia, and 0.178 for Thailand, indicating that the complete set of predictors accounted for 39.9% of the variance in CCI among the overall SMT sample.

In summary, across the new generations of ethnic Chinese in SMT, Chinese content exposure (H1a) and visits to China (H2a) emerged as the two factors consistently and positively associated with CCI across the overall SMT sample and all three individual country sub-samples. These relationships held robustly regardless of national context. However, large screen preference (RQ1) showed a significant association only in the overall sample but not consistently across the three countries, while Chinese language proficiency (H2b) demonstrated mixed results across countries. The final model explained 39.9% of the variance in CCI among the new generations of ethnic Chinese in SMT, with country-specific explanatory power ranging from 17.8% (Thailand) to 34.8% (Malaysia). These findings suggest that among the new generations of ethnic Chinese in SMT, those who demonstrate higher levels of Chinese content exposure and more frequent visits to China are more likely to identify with Chinese culture.

### 4.5 Mediating roles of Chinese content exposure

This section examines the mediating role of Chinese content exposure in the relationships between three predictor variables-large screen preference, visits to China, and Chinese language proficiency-and CCI among the new generations of ethnic Chinese in SMT.

As shown in Table 8, regarding the mediating role of Chinese content exposure in the relationship between large screen preference and CCI, the analysis revealed mixed patterns. In the overall SMT sample, the indirect effect of large screen preference on CCI through Chinese content exposure was negative and significant (*B* = −0.11, 95% CI [−0.17, −0.05], *p* < 0.01), while the total effect was not significant (*B* = −0.02, 95% CI [−0.08, 0.05], *p* > 0.05). However, when examined across the three countries, this pattern was not consistently replicated. In Singapore, the indirect effect was negative and significant (*B* = −0.16, 95% CI [−0.26, −0.06], *p* < 0.01). Also, in Malaysia, the indirect effect was negative and significant (*B* = −0.19, 95% CI [−0.28, −0.10], *p* < 0.01). In Thailand, the indirect effect (*B* = −0.02, 95% CI [−0.06, 0.01]) did not reach statistical significance. Given that the indirect effect of Chinese content exposure between large screen preference and CCI was significant only in the overall SMT sample and in Singapore and Malaysia, but not in Thailand, this mediating relationship is not considered consistently supported across all three countries. H3a is therefore rejected.

**TABLE 8:** The mediating effects of Chinese content exposure.

Impact pathway	SMT (*N* = 1,298)			Singapore (*N* = 424)			Malaysia (*N* = 432)			Thailand (*N* = 442)		
	Direct effects	Indirect effects	Total effects	Direct effects	Indirect effects	Total effects	Direct effects	Indirect effects	Total effects	Direct effects	Indirect effects	Total effects
LSP→ CCE→ CCI	0.09* [0.04, 0.15]	−0.11**[−0.17, −0.05]	−0.02 [−0.08, 0.05]	0.10 [−0.02, 0.21]	−0.16** [−0.26, −0.06]	−0.06 [−0.20, 0.07]	0.07 [−0.05, 0.19]	−0.19** [−0.28, −0.10]	−0.12 [−0.25, 0.01]	0.03 [−0.03, 0.09]	−0.02 [−0.06, 0.01]	0.01 [−0.06, 0.07]
VTC→ CCE→ CCI	0.46** [0.38, 0.54]	0.31** [0.26, 0.36]	0.77** [0.70, 0.85]	0.28** [0.10, 0.46]	0.36** [0.25, 0.48]	0.64** [0.47, 0.80]	0.48** [0.34, 0.62]	0.29** [0.21, 0.36]	0.77** [0.64, 0.90]	0.29** [0.18, 0.40]	0.10** [0.03, 0.19]	0.39** [0.30, 0.49]
CLP→ CCE→ CCI	0.23** [0.20, 0.27]	0.14** [0.12, 0.16]	0.37** [0.34, 0.41]	0.13** [0.05, 0.20]	0.14** [0.09, 0.18]	0.27** [0.18, 0.35]	0.24** [0.16, 0.32]	0.11** [0.07, 0.15]	0.35** [0.26, 0.44]	0.11** [0.04, 0.17]	0.06** [0.03, 0.11]	0.17** [0.11, 0.24]

LSP, Large screen preference; VTC, Visits to China; CLP, Chinese language proficiency; CCE, Chinese content exposure; CCI, Chinese cultural identity.

**Indicates significant relation at the 0.01 level; *indicates significant relation at the 0.05 level; the values in brackets are 95% confidence intervals.

The mediating role of Chinese content exposure in the relationship between visits to China and CCI showed strong and consistent support. In the overall SMT sample, the indirect effect through Chinese content exposure was positive and significant (*B* = 0.31, 95% CI [0.26, 0.36], *p* < 0.01). This pattern of positive and significant mediating effects was consistently observed across all three countries. In Singapore, the indirect effect was positive and significant (*B* = 0.36, 95% CI [0.25, 0.48], *p* < 0.01). In Malaysia, the indirect effect was also positive and significant (*B* = 0.29, 95% CI [0.21, 0.36], *p* < 0.01). In Thailand, the indirect effect was positive and significant (*B* = 0.10, 95% CI [0.03, 0.19], *p* < 0.01). This indicates that higher levels of visits to China are associated with greater Chinese content exposure, which in turn is associated with stronger CCI. Thus, the H3b is supported.

**FIGURE 2:**
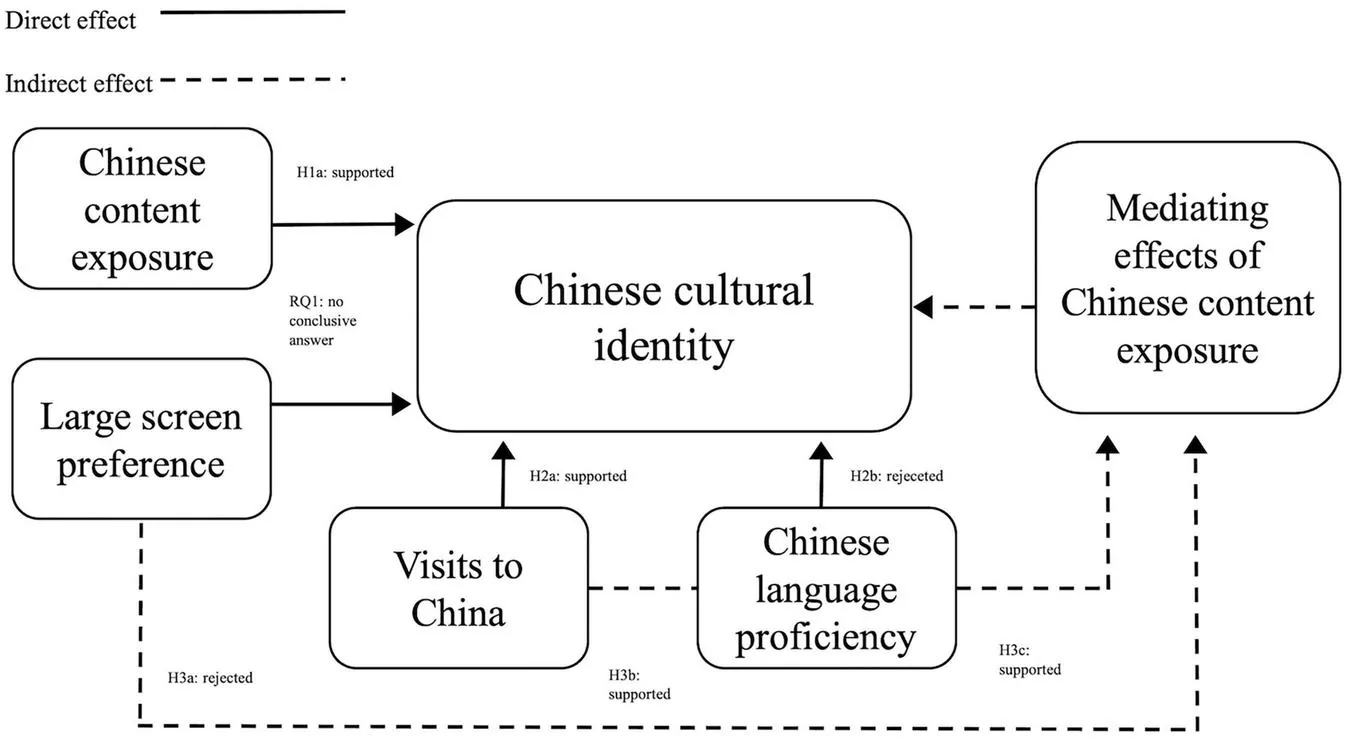
Hypothesis test results.

The mediating role of Chinese content exposure in the relationship between Chinese language proficiency and CCI showed consistent patterns across countries. In the overall SMT sample, the indirect effect through Chinese content exposure was positive and significant (*B* = 0.14, 95% CI [0.12, 0.16], *p* < 0.01). When examined across the three countries, this pattern was also consistently replicated. In Singapore, the indirect effect was positive and significant (*B* = 0.14, 95% CI [0.09, 0.18], *p* < 0.01). In Malaysia, the indirect effect was also positive and significant (*B* = 0.11, 95% CI [0.07, 0.15], *p* < 0.01). The same in Thailand, the indirect effect was positive and significant (*B* = 0.06, 95% CI [0.03, 0.11], *p* < 0.01). This indicates that higher levels of Chinese language proficiency are associated with greater Chinese content exposure, which in turn is associated with stronger CCI. Therefore, H3c is supported. The results of all hypothesis tests are presented in Figure 2.

## 5 Conclusion and discussion

### 5.1 The CCI among new generations of ethnic Chinese in SMT

Malaysian new-generation ethnic Chinese exhibit the highest levels of CCI, Chinese language proficiency, and visits to China. From the mid-nineteenth century onward, British colonial authorities introduced large-scale Chinese and Indian labor into Malaysia to meet the demands of tin mining and rubber plantation development, fundamentally transforming the region’s ethnic composition and creating a tri-ethnic structure of Malays, Chinese, and Indians (Jackson, 1961). Crucially, the British adopted a “divide and rule” policy. Politically, they administered Malays through Sultans, Chinese through Kapitan Cina and the Chinese Protectorate, and Indians through separate labor institutions (Abraham, 1983). This colonial strategy not only severed inter-ethnic economic and social ties but also created significant inter-ethnic economic disparities, generating Malay distrust toward the Chinese as potential economic threats (Manickam, 2009). After gaining independence, Malaysia has maintained a distinctive ethnic political character, with the Malay-dominated ruling coalition consistently prioritizing “Ketuanan Melayu” (Malay supremacy) (Zhang, 2024). These policies, while aiming to narrow inter-ethnic economic disparities, have systematically disadvantaged Malaysian Chinese in education and employment. At the same time, it has exacerbated tensions among different ethnic groups. In response to these exclusionary policies, the Chinese community has mounted vigorous defensive actions to preserve its cultural and educational institutions, including the Independent Secondary School Revival Movement, the defense of cultural heritage sites, and the establishment of the National Chinese Cultural Festival (Yi, 2020). As a result, Malaysia has become the country outside China that maintains a complete Chinese education system from primary through secondary to tertiary levels. Chinese education in Malaysia has been sustained through the collective efforts of Chinese community organizations, Chinese-language media, and generations of educators who have treated Chinese schools as the “spiritual pillar and root of identity” for the Chinese community (Tan, 2011). This institutionalized educational infrastructure ensures that Malaysian Chinese youth receive systematic exposure to the Chinese language and Chinese cultural content from an early age, which in turn reinforces their Chinese cultural identity. Also, Malaysian Chinese actively seek opportunities for “visits to China” as a means of reconnecting with ancestral heritage. Research has documented that for Sarawakian Chinese, such visits represent a “reality of return” that reinforces cultural identification (Tie et al., 2015).

As for Singapore, it is the only country outside China with a Chinese-majority population. However, its new-generation ethnic Chinese showed high levels of Chinese content exposure, but lower levels of CCI and Chinese language proficiency than their Malaysian counterparts. This situation of Singaporean Chinese is understandable from political perspective. Singapore’s foreign policy is fundamentally shaped by its geopolitical vulnerability as a “little red dot in a green ocean” (Robyn, 2012). Therefore, Singapore has adopted a deliberate hedging strategy in its foreign policy, maintaining strong ties with both the United States and China while avoiding alignment with either. Singapore engages deeply with both powers, participating in China’s Belt and Road Initiative (BRI) while also joining the US-led Indo-Pacific Economic Framework for Prosperity (IPEF) (Min, 2025). In security terms, Singapore prefers to have the United States as an offshore balancing power against China’s rise (Gao and Wang, 2019). The imperative of regional balancing constrains the extent to which CCI can be politically mobilized or prioritized in Singapore. In terms of Singapore’s domestic policy, though Singapore is also made up of three major ethnic groups-Chinese, Malay, and Indian-the ruling People’s Action Party (PAP) has consistently sought to de-emphasize ethnic differences, governing on principles of pragmatism and secularism, with the philosophy that government should “help those who are willing to put in effort” (Gao and Wang, 2019). Therefore, ethnic tensions in Singapore are relatively more moderate. Moreover, as ethnic Chinese constitute the majority population in Singapore, they may not perceive a strong need to reinforce their CCI as a defensive response to other ethnic groups, which led to their relatively lower level of CCI compared to their counterparts in Malaysia. Also, while Singapore has a structured Chinese-language education system, English has become the dominant language in nearly every domain of everyday social life among Chinese Singaporeans (Goh and Fong, 2021). Research indicates that while Chinese Malaysians continue to perceive Chinese as the language most important to Chinese ethnicity, but English has achieved the dominant position in most aspects of daily life (Wong, 2016). This linguistic shift, reinforced by Singapore’s bilingual education policy, has contributed to its declining Chinese language proficiency.

The finding that Thai new-generation ethnic Chinese exhibit the lowest levels across three variables (namely CCI, Chinese language proficiency, and visits to China) is consistent with Thailand’s long history of assimilationist policies toward its Chinese population and its unique status as the only Southeast Asian country never colonized (Yi and Sun, 2022). Thailand’s avoidance of colonial subjugation granted its “governance autonomy,” which is the capacity to proactively regulate immigration and shape the integration of Chinese migrants according to national development needs. During the critical historical period of large-scale Chinese migration, Thailand was able to independently determine immigration policy, control the scale of Chinese immigration, moderate Chinese nationalist sentiment, and actively channel Chinese capital into national development (Yi and Sun, 2022). In order to assimilate the Chinese, the Thai government mandated Thai-language education, and also implemented a surname-change policy, requiring ethnic Chinese to adopt Thai surnames (Lang and Numtong, 2026). The disruption of Chinese-language education created a generational gap in Chinese language proficiency, with those under fifty often unable to speak or read Chinese. As a result of these historical trajectories, Thai Chinese have undergone thorough process of “localization” among Southeast Asian Chinese communities. Many have chosen complete assimilation and have gradually lost connection with their ancestral homeland (Yi and Sun, 2022). Therefore, the new generations of ethnic Chinese in Thailand have the relatively lowest level of CCI, Chinese language proficiency, and visits to China among the three countries.

### 5.2 The factors associated with CCI among new generations of ethnic Chinese in SMT

This study also examined the factors associated with CCI among the new generations of ethnic Chinese in SMT. The hierarchical regression analysis revealed that, across the overall SMT sample and consistently across all three country-specific sub-samples, Chinese content exposure and visits to China emerged as the two factors most robustly and positively associated with CCI. In contrast, large screen preference showed a significant association only in the overall sample but not consistently across the three countries, while Chinese language proficiency demonstrated mixed results, being significant only in Malaysia but not in Singapore or Thailand.

Firstly, Chinese content exposure appears to be a key factor associated with CCI in our model. This finding aligns with a growing body of research on the role of media in diasporic identity formation (Peng and Xie, 2022; Liu et al., 2023; Loi, 2023). Recent observations indicate that Chinese social media platforms have experienced rising usage rates across Southeast Asia (Peng, 2025), particularly in markets with substantial Chinese populations such as Singapore, Malaysia, Indonesia, and Thailand. These platforms have been described as constituting a digital Chinatown, which is a virtual space where overseas Chinese exchange information, support one another, and sustain cultural memory, functions historically performed by physical China towns. Notably, this phenomenon has extended beyond Chinese-speaking users to broader international audiences, as everyday interactions around food, pets, urban life, and healthcare facilitate genuine grassroots cultural engagement. The consistency of this finding across Singapore, Malaysia, and Thailand suggests that Chinese content exposure functions as a relatively universal mechanism for cultural identity reinforcement among overseas Chinese, irrespective of national context.

Also, visits to China are consistently and positively associated with CCI. The results also align with a substantial body of literature documenting the positive associations between inter-ethnic contact and cultural identity among diaspora communities (Wang et al., 2024; Li et al., 2025). This can be understood through the lens of inter-group contact theory, which posits that meaningful, cooperative, and institutionally supported cross-group interactions foster mutual understanding, reduce inter-group anxiety, and ultimately strengthen identification with one’s heritage culture (Pettigrew et al., 2011). In the SMT context, visits to China provide rich opportunities for sustained and meaningful engagement with Chinese cultural practices, thereby reinforcing CCI.

Furthermore, the finding that large screen preference showed a significant association with CCI in the overall SMT sample but not consistently across the three individual countries warrants careful interpretation. This inconsistency may reflect the fact that “large screen preference” is a relatively novel and under-explored construct in the context of diasporic cultural identity. The relationship between large screen preference and CCI remains to be further explored in future work.

Finally, the finding that Chinese language proficiency was significantly associated with CCI only in Malaysia, but not in Singapore or Thailand, represents a notable point of divergence from the existing literature. Prior research has established that language plays a vital function in shaping CCI. However, the findings of the present study suggest that the relationship between Chinese language proficiency and CCI needs to be examined on a country-by-country basis. In Malaysia, Chinese Malaysians face structural pressures that reinforce the importance of heritage language maintenance. Chinese language retains a central role in cultural identity construction (Yi, 2020). In Singapore, by contrast, English has become the dominant language in nearly every domain of everyday social life among Chinese Singaporeans (Goh and Fong, 2021). As a result, Chinese language proficiency no longer serves as a reliable marker of CCI in the Singaporean context, in which one can identify strongly with Chinese culture without being highly proficient in the language. In Thailand, the historical assimilation of the Chinese population into Thai society has meant that Chinese language proficiency has been largely lost across generations (Yi and Sun, 2022). Many Thai Chinese of the younger generation are unable to speak or read Chinese, yet this does not necessarily preclude some degree of cultural identification. The present findings suggest, however, that in the Thai context, Chinese language proficiency is not a significant correlate of CCI, likely because cultural identity among Thai Chinese is now more strongly influenced by engagement with China-related media, rather than by active language competence.

### 5.3 The mediating roles of Chinese content exposure

This study also tests the mediating effects of Chinese content exposure. First, the mediating role of Chinese content exposure in the relationship between large screen preference and CCI yielded mixed and somewhat unexpected results. An intriguing and somewhat counter-intuitive finding in this analysis is the negative indirect effect of large screen preference on Chinese content exposure, observed in the overall SMT sample as well as in Singapore and Malaysia, though not in Thailand. This suggests that individuals who prefer large screens tend to consume less Chinese content specifically. Several possible explanations may account for this unexpected finding. First, large screens, such as television sets, home theater systems, and digital cinema screens, are often associated with different content genres and platforms than small screens. While large screens are commonly used for mainstream entertainment, such as Hollywood films, sports broadcasts, and popular television series, Chinese content is increasingly consumed on mobile devices through streaming platforms, social media apps (such as TikTok, WeChat, and Xiaohongshu), and video-sharing websites (Peng, 2025). This digital platform ecology means that audiences who rely heavily on large screens may be less exposed to the China-related content that circulates primarily through mobile-first platforms. Secondly, large screen viewing is often a social or family activity, such as watching films or television together in shared spaces. This social context may shape content choices toward what is acceptable or appealing to a broader audience, which in multilingual households may not be Chinese-language content. By contrast, small-screen viewing on personal devices is typically more individual and private, allowing for more niche, interest-driven consumption that may include Chinese-language content. Thirdly, the measurement scale used in this study may not have been fully mature, which could have influenced the results. Future work could develop a more robust scale for large screen preference.

The mediating role of Chinese content exposure in the relationship between visits to China and CCI showed strong and consistent support across all three countries. These findings suggest that visits to the ancestral homeland not only have a direct positive effect on CCI but also exert an indirect effect by increasing individuals’ consumption of Chinese media content. When overseas Chinese visit China, they may be exposed to Chinese media recommendations, cultural events, and shared content, thereby increasing their engagement with Chinese cultural materials upon their return.

Also, the mediating role of Chinese content exposure in the relationship between Chinese language proficiency and CCI showed consistent support across all three countries. These findings indicate that higher levels of Chinese language proficiency are associated with greater Chinese content exposure, which in turn is associated with stronger CCI. This result is consistent with prior research demonstrating that heritage language proficiency serves as a key marker of cultural identity and a conduit for cultural transmission (Wang, 2023; Zhou and Liu, 2023). Language proficiency lowers the barriers to media consumption, enabling deeper and more frequent engagement with Chinese cultural content, and then enhance the CCI.

## 6 Practical implications

The findings of this study suggest several avenues for practice that may be considered by relevant stakeholders.

For educational institutions and cultural organizations, the consistent association between Chinese content exposure and CCI across all three countries suggests that facilitating access to diverse, engaging China-related media content, including films, television programs, and publications, may be a meaningful way to support cultural identification among overseas Chinese youth. Rather than focusing exclusively on formal language instruction, which showed inconsistent associations, practitioners might consider integrating media-based approaches that allow young people to encounter Chinese culture in everyday, relatable contexts.

For organizations involved in heritage tourism and exchange programs, the robust association between visits to China and CCI underscores the potential value of facilitating meaningful engagement with the ancestral homeland. Programs such as “root-seeking” tours, educational exchanges, and family heritage visits may provide overseas Chinese youth with opportunities for direct cultural immersion that complement media-based exposure. However, the design of such programs warrants careful consideration. Visits that allow for genuine interaction, personal exploration, and emotional connection may be more effective than those that are purely touristic or ceremonial.

For media producers and content creators, the findings suggest that China-related media content that resonates with overseas Chinese audiences may play a meaningful role in sustaining cultural connections. The emergence of digital China towns on Chinese social media platforms points to the potential of interest-based communities to function as spaces of cultural connection that transcend geographic boundaries.

For policymakers and community leaders, the cross-national variations observed in this study suggest that approaches to supporting CCI among overseas populations need to be sensitive to the specific historical, educational, and linguistic contexts of each country. What works in Malaysia, where a robust Chinese educational infrastructure already exists, may not be appropriate or effective in Thailand, where the historical trajectory of assimilation has created a very different cultural landscape. A one-size-fits-all approach is unlikely to be optimal.

Finally, several limitations of this study merit acknowledgment. First, the sample consists mainly of online-recruited respondents, which may introduce self-selection bias. Second, the use of English as the survey language may have introduced a bias toward Thai Chinese respondents with higher English proficiency, potentially under-representing those with limited English skills. Although we argue that English proficiency is generally unrelated to CCI, this sampling constraint nonetheless limits the generalizability of our findings to the broader Thai Chinese population. Third, the AVE values for Chinese content exposure and large screen preference fell below the recommended threshold of 0.50, suggesting that these constructs may not have been optimally operationalized. Future research could develop more refined and validated instruments for assessing screen preference and media exposure in diaspora contexts. Fourth, the lack of official demographic benchmarks for the new generations of ethnic Chinese constrained our ability to fully assess the representativeness of the sample. Fifth, the cross-sectional design of this study precludes causal inferences; the observed associations and mediation effects should be interpreted as correlational rather than causal. Longitudinal or experimental designs would be valuable in future research to establish temporal ordering and causal direction. Despite these limitations, the present study offers a systematic and integrative examination of the factors shaping CCI among new-generation ethnic Chinese in SMT, and provides a foundation for future research to build upon.

## Data Availability

The original contributions presented in the study are included in the article/supplementary material, further inquiries can be directed to the corresponding author.
